# Effect of nano-zinc oxide on doxorubicin- induced oxidative stress and sperm disorders in adult male Wistar rats

**Published:** 2013-05

**Authors:** Puran Badkoobeh, Kazem Parivar, Seyed Mehdi Kalantar, Seyed Davood Hosseini, Alireza Salabat

**Affiliations:** 1 *Department of Biology, Science and Research Branch, Islamic Azad University, Tehran, Iran.*; 2 *Research and Clinical Center for Infertility, Shahid Sadoughi University of Medical Sciences, Yazd, Iran.*; 3 *Razi Vaccine and Serum Research Institute, Central Area Branch, Arak, Iran.*; 4 *Department of Chemistry, Arak University, Arak, Iran.*

**Keywords:** *Doxorubicin*, *Antioxidant*, *DNA damage*, *Lipid peroxidation*, *Spermatogenesis*, *Testosterone*

## Abstract

**Background: **Doxorubicin (DOX), an anthracycline antibiotic, is a widely used anticancer agent. In spite of its high antitumor efficacy, the use of DOX in clinical chemotherapy is limited due to diverse toxicities, including gonadotoxicity.

**Objective:** We investigated the protective effect of nano-zinc oxide (nZnO) as an established antioxidant on DOX-induced testicular disorders.

**Materials and Methods: **In this experimental study 24 adult male Wistar rats were divided into four groups including one control and three experimentals (6 rats per group). They received saline (as control), DOX alone (6 mg/kg body weight, i.p.), nZnO alone (5 mg/kg body weight, i.p.), and nZnO followed by DOX. Animals were sacrificed 28 days after treatment and evaluations were made by sperm count and measuring sex hormone levels in plasma. Also total antioxidant power (TAP) and lipid peroxidation (LPO) in plasma were tested. Data was analyzed with SPSS-14 and one way ANOVA test. P<0.05 were considered to be statistically significant.

**Results:** In the DOX-exposed rats significant differences were found compared with the control group (p=0.001) in plasma total antioxidant power (TAP) (425.50±32.33 vs. 493.33±18.54 mmol/mL), Lipid peroxidation (LPO) (3.70±0.44 vs. 2.78±0.68 μmol/mL), plasma testosterone (3.38±0.69 vs. 5.40±0.89 ng/dl), LH (0.26±0.05 vs. 0.49±0.18 mlU/mL), sperm count (157.98±6.29 vs. 171.71±4.42×10^6^/mL) and DNA damage (11.51±3.45 vs. 6.04±2.83%). Co-administration of nZnO significantly improved DOX-induced changes (p=0.013) in plasma TAP (471.83±14.51 mmol/mL), LPO (2.83±0.75 μmol/mL), plasma testosterone (5.00±1.07 ng/dl), LH (0.52±0.08 mlU/mL), sperm count (169.13±5.01×10^6^/mL) and DNA damage (7.00±1.67%).

**Conclusion:** At the dose designed in the present investigation cytoprotective role of nano-zinc oxide through its antioxidant potential is illuminated in DOX-induced male gonadotoxicity.

## Introduction

The anthracycline antibiotic adriamycin, or doxorubicin (DOX), is a well-known anticancer drug in treatment of various tumors, although one of its adverse effects is male infertility (1). DOX exhibits toxicity to the reproductive system, disturbing male fertility (2). Though gonadal damage by antineoplastic drugs like DOX, is commonly observed, but it has been relatively less studied compared to the other possible side effects (3). Zanetti *et al* showed that treatments with DOX cause an important decrease in testicular weight resulting to reduced spermatogenic cell number (1). A significantly decreased number of sperm and low sperm motility were also observed in rats (4, 5). 

In addition, thereis an increased apoptosis in spermatogonia and spermatocytes following DOX treatment (6). Induction of apoptosis by this drug is one of the earliest signs of genotoxic damage to the adult testis. But the mechanisms by which this chemotherapeutic agent damages spermatogenesis, is little known (7). Saalu *et al* reported that DOX produces persistent damage to the spermatogenic cells as well as increase in the testicular oxidative stress (8). DOX toxicity can be mediated by interaction with topoisomerase II, an enzyme that is abundant in cells with rapid proliferation (9). 

The cytotoxic or apoptotic action of DOX may also be exerted by other mechanisms such as membrane composition differentiation and function alteration, DNA binding or oxygen free radicals formation (5, 10). Although a number of possible toxic mechanisms have been recognized following exposure to DOX, the main pathogenic mechanism appears to involve the generation of reactive oxygen species (ROS) (4, 11). 

Some experimental studies have shown that ROS are important agents for tissue damage (12). It has been demonstrated that oxygen radical-induced damage of lipids in membrane is the key factor for DOX-induced toxicity (13). These processes also occur in the testis of DOX-treated animals, elucidating the high susceptibility of proliferating germ cells and directing to pretreatment with antioxidants as a promising form of reducing DOX toxicity (1). 

On the other hand, the ability of zinc to delay oxidative processes has been documented for many years (14). Zink is known to be a protecting antioxidant as it is able to bring the Malon dialdehyde (MDA) levels near normal levels (15, 16). Zinc acts as a cofactor for more than 80 enzymes involved in DNA transcription and protein synthesis. DNA transcription is a main part of germ cell development therefore zinc may be important for reproduction (17). 

Nano-Zinc oxide (nZnO) is a new product with particle diameter between 1-100 nm (18, 19). Recently, nZnO has been considered as an important factor in the area of animal science (18). It appears that nano materials hold excessive potential to pass some of the barriers to efficient targeting of cells and molecules in many diseases (10, 20). Still further studies are needed in order to find the mechanism of the nano material defensive effects.

Therefore, the present study was designed to examine the protective effect of nZnO on antioxidant power, sex hormone levels and sperm characteristics following exposure to DOX in male Wistar rats.

## Materials and methods

**Chemicals**

DOX (Ebewe Pharma co. Austria), nZnO (Sigma-Aldrich, particle size<50 nm [TEM], purity >97%), Ham’sF10, NaHCO3, eosin-Y, ethanol, formalin, hematoxylin, paraffin, Carnoy’s fixative (methanol/Acetic acid; 1/3), glutaraldehyde, acridine orange, 1,1,3,3,-tetra ethoxy-propane, trichloroacetic acid, n-butanol, 2,4,6-tripyridyl-s-triazine (TPTZ), 2- thiobarbituric acid (TBA), acetic acid, and phosphate buffer (Merck Chemical Co. Darmstadt, Germany) were used in this study.

**Preparation of nZnO suspension**

NZnO particles were suspended in 1% sdium carboxy methyl cellulose as stabilizer or surfactant, stirred with magnetic stirrer for 5 minutes and then dispersed by ultrasonic vibration for 15 min (21-23). In order to avoid the aggregation of the particles fresh suspension was prepared before every use.

**Animal treatments**

In this experimental study, 24 adult sexually mature male (4 months old weighing 220-250 g) Wistar rats were obtained from Razi Vaccine and Serum Research Institute (Tehran). They were kept under standard conditions of temperature (23±2^o^C), and 12h light/dark period, and fed with a standard pellet diet and water ad libitum. Animal handling and care were performed in accordance with the guidelines established by the Canadian Council on Animal Care. In this study, four groups each containing six male rats were used. 

Treatment groups were as follows: group 1 received normal saline by injection (ip) daily, group 2 received DOX (6 mg/kg/day) dissolved in normal saline, group 3 received nZnO (5 mg/kg/day) dissolved in normal saline by ip injection, and group 4 received DOX (6 mg/kg/day) and nZnO (5 mg/kg/day) following pretreatment with nZnO one day before. All groups were treated for 3 days. 

**Sampling**

After 28 days, the animals were euthanized by CO_2_ exposure and were killed by decapitation. Blood samples were collected in vials containing heparin. 

The plasma was separated and kept at -80^o^C until analysis of LH, FSH, testosterone, and toxic stress markers including cellular lipid peroxidation (LPO) and total antioxidant power (TAP). Epididymes were removed, cleaned of adhering connective tissue, weighed and perfused with cold (0.9%) NaCl. Radioimmunoassay kits were used to determine concentrations of LH, FSH, and testosterone. The study was approved by the ethic committee of the Razi Institute. 

**LPO and TAP**

Concentration of LPO in plasma was determined by measurement of malonedialdehyde and other lipid peroxide aldehydes that react with TBA known as TBA-reactive substances (TBARS). The absorption of the TBARS was deterImined spectrophotometrically at 532 nm using 1, 1, 3, 3-tetraethoxypropan as standard (24). TAP of plasma and testis was determined by measuring their ability to reduce Fe^3+^ to Fe^2+^. The complex between Fe^2+^ and TPTZ gives a blue color with absorbency at 593 nm (25). 

**Sperm characteristics**

Epididymal sperms were collected by slicing the epididymes in 5 mL of Ham’s F10 and incubating for 5 min at 37^o^C in an atmosphere of 5% CO_2_ to allow sperm to swim out of the epididymal tubules. One drop of sperm suspension was placed on a microscope slide, and a cover slip was placed over the droplet. At least 10 microscopic fields were observed at 400× magnification using a phase contrast microscope, and the percentage of motile sperm was evaluated microscopically within 2-4 min of their isolation from the epididymes and was considered as a percentage of motile sperm of the total sperm counted. 

Epididymal sperm counts were obtained by the method described in the WHO Manual (1999). Briefly, 5 μl aliquot of epididymal sperm was diluted with 95 μl of diluent (0.35% formalin containing 5% NaHCO_3_ and 0.25% trypan blue) and approximately 10 μl of this diluted specimen was transferred to each of the counting chambers of the hemocytometer and was allowed to stand for 5 min in a humid chamber to prevent drying. The cells sediment during this time and were counted with a light microscope at 400× (26). 

A 20 μl of sperm suspension was mixed with an equal volume of 0.05% eosin-Y. After 2 mins incubation at room temperature, slides were viewed by bright-field microscope with magnification of 400×. Dead sperms appeared pink and live sperms were not stained. Two hundred sperms were counted in each sample and viability percentages were calculated. For analysis of morphological abnormalities, sperm smears were drawn on clean and grease-free slides, and allowed to air dry overnight. The slides were stained with 1% eosin-Y/5% nigrosin and examined at 400× for morphological abnormalities such as amorphous, bicephalic, coiled, or abnormal tails (26, 27). 

**Staining of spermatozoa with acridine orange**

Acridine orange staining was used to monitor the effects of DOX on cauda epididymal sperm. To perform this assay with fluorescent microscope, thick smears were fixed in Carnoy’s fixative (methanol: acetic acid 1: 3) for at least 2 h. The slides were stained for 5 min and gently rinsed with deionized water. Two-hundred sperms from each staining protocol were evaluated and graded as normal DNA (green) or damaged DNA (yellow to red) (28). 

**Sample preparation for light microscopy and histopathological analysis**

After fixation of epididymes in a 10% formalin solution, they were directly dehydrated in a graded series of ethanol and embedded in paraffin. Thin sections (4-5 μm) were cut using a microtome and stained with hematoxylin and eosin and examined using a light microscope. The qualitative changes of epididymes were recorded (26). 

**Statistical analysis**

Values are reported as mean±SEM. Statistical significance between groups was computed by analysis of variance and Tukey multiple comparison post hoc tests. Data was analyzed with SPSS-14 and one way ANOVA test. P<0.05 was considered significant.

## Results

Treatment with DOX showed no difference on the survival and behavior of the animals. Administration of DOX significantly increased plasma LPO as compared to control (3.70±0.44 vs. 2.78±0.68 μmol/mL, p=0.018). Co-administration of DOX and nZnO resulted in restoration of DOX-induced LPO changes in plasma of DOX-treated animals (2.83±0.75 μmol/mL, p=0.027) (Table I). 

TAP was decreased in plasma of DOX-treated animals as compared to control (425.50±32.33 vs. 493.33±18.54 mmol/mL, p=0.000). Coadministration of DOX and nZnO restored DOX-induced reduction of TAP in plasma of DOX-treated animals (471.83±14.51 mmol/mL, p=0.013) (Table I). The weight of testis significantly decreased as compared to control (1.27±0.15 vs. 1.69±0.22g, p=0.005). 

In nZnO+DOX group testis weight significantly increased compared to DOX-exposed group (1.66±0.15g, p=0.009). Epididymes weight and FSH levels showed no significant difference between groups (Table II). Administration of DOX decreased plasma LH (0.26±0.05 vs. 0.49±0.18 mlU/mL, p=0.021) and testosterone concentrations compared to control (3.38±0.69 vs. 5.40±0.89 ng/dl, respectively p=0.005). 

Co-administration of DOX and nZnO recovered DOX-induced reduction of plasma LH (0.52±0.08 mlU/mL, p=0.010) and testosterone (5.00±1.07 ng/dl, p=0.027) (Table II). Treatment of male rats with DOX resulted in reduced sperm count (157.98±6.29 vs. 171.71±4.42 10^6^/mL, p=0.002), while the number of dead (20.67±2.7 vs. 38.83±3.31%, p=0.000) and abnormal sperms (15.17±1.47 vs. 6.00±1.26%, p=0.000) increased (Table II). All changes were restored by nZnO cotreatment (14.67±2.94%, p=0.000, 9.83±1.17%, p=0.000 respectively) (Table II). 

**Figure 1:**
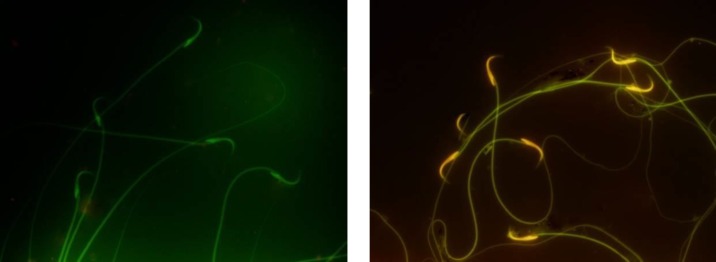
Acridine orange staining of spermatozoa. Green sperms show normal DNA (control group), while yellow to red sperms show damage to DNA (DOX-exposed group

In DOX-treated group percentage of DNA damage in the epididymal sperm significantly increased compared to control group (11.51±3.45 vs. 6.04±2.83%, p=0.009). nZnO blocked DOX-induced DNA damage (7.00±1.67%, p=0.038) (Table II, Figure 1).

**Histopathological findings**

**Figure 2:**
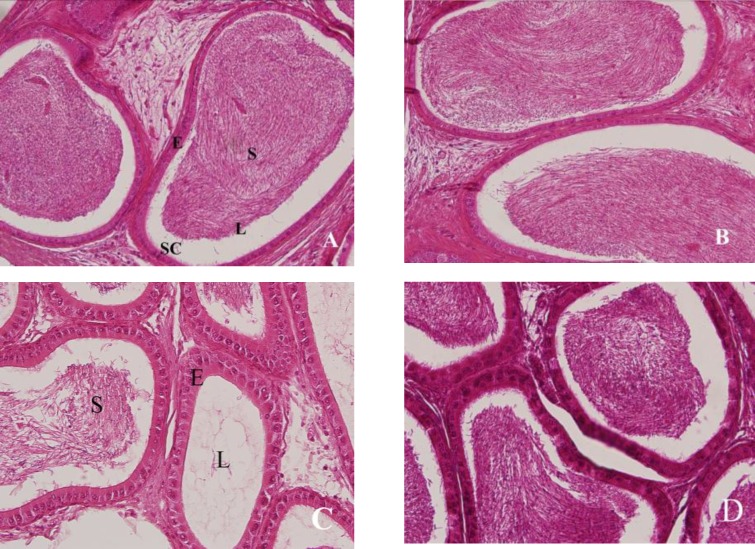
Photomicrographs of cauda epididymis showing sperm density in the lumina of tubular sections, (H & E, ×40). Epididymes from control (A) and nano-Zinc Oxide (B) rats show normal architecture of tubules and intertubular elements. Epithelium is pseudostratified, columnar and the lumen contains densely-packed mature spermatozoa. Epididymal tubules of Doxorubicin-exposed (C) rats reveal damaged epithelium and decreased tubular size. Note that sperm density is severely decreased. Some tubules are empty and others have few spermatozoa. Administration of nano-Zinc Oxide along with doxorubicin (D) improved these changes. Sperm density slightly decreased. L: lumen, E: epithelium, SC: stereocilia, S: sperm.

Epididymis of the control and nZnO-treated rats showed normal histological structure with normal sperm density. Large tubules lined by pseudostratified columnar epithelium with abundant stereocilia projection from their surface. Long and dense processes of sperm tails were observed within the lumina (Figure 2-A, B). The epididymal tubules of DOX-exposed rats were smaller and the majorities of them were free from mature spermatozoa. Epithelium contained numerous number of sloughed cells with cell debris in lumina (Figure 2-C). Epididymes of DOX+nZnO-treared rats showed normal architecture of tubules and intertubular elements. Density of spermatozoa in tubules showed slight decrease when compared to control group (Figure 2-D).

**Table I:** Effects of DOX and nZnO on oxidative stress biomarkers in plasma of male Wistar rats

	**Control**	**DOX**	**nZnO**	**DOX + nZnO**
Plasma LPO (μmol/mL)	2.78 ± 0.68	3.70 ± 0.44^a^	1.97 ± 0.35^a,b^	2.83 ± 0.75^b^
Plasma TAP (mmol/mL)	493.33 ± 18.54	425.50 ± 32.33^a^	571.17 ± 15.51^a,b^	471.83 ± 14.51^b^

nZno: nano-Zinc oxide           DOX: Doxorubicin          LPO: lipid peroxidation           TAP: total antioxidant power

Data are expressed as mean±S.D.

^ a^ Significantly different between control and other groups at p<0.05 using scheffe test.

^b^ Significantly different between DOX and other groups at p<0.05 using scheffe test.

**Table II:** Effects of DOX and nZnO on the organ weight, sex hormone levels, and sperm characteristics of male Wistar rats

	**Control**	**DOX**	**nZnO**	**DOX + nZnO**
Testis weight (g)	1.69 ± 0.22	1.27 ± 0.15^a^	1.83 ± 0.15^b^	1.66 ± 0.15^b^
Epididymal weight (g)	0.51 ± 0.15	0.43 ± 0.06	0.63 ± 0.09^b^	0.48 ± 0.09
FSH (mlU/mL)	4.18 ± 0.43	3.17 ± 1.14	4.78 ± 1.38	3.75 ± 0.61
LH (mlU/mL)	0.49 ± 0.18	0.26 ± 0.05^a^	0.77 ± 0.12^a,b^	0.52 ± 0.08^b^
Testosterone (ng/dl)	5.40 ± 0.89	3.38 ± 0.69^a^	6.07 ± 0.59^b^	5.00 ± 1.07^b^
Sperm count (10^6^/mL)	171.71 ± 4.42	157.98 ± 6.29^a^	174.16 ± 4.18^b^	169.13 ± 5.01^b^
Motility (%)	77.83 ± 5.49	74.67 ± 5.95	80.83 ± 1.47	72.17 ± 6.31
Dead sperms (%)	8.83 ± 3.31	20.67 ± 2.73^a^	7.33 ± 1.63^b^	14.67 ± 2.94^a,b^
Abnormal sperms (%)	6.00 ± 1.26	15.17 ± 1.47^a^	5.33 ± 0.82^b^	9.83 ± 1.17^a,b^
Positive acridine orange staining (%)	6.04 ± 2.83	11.51 ± 3.45^a^	2.50 ± 1.05^b^	7.00 ± 1.67^b^

nZno: nano-Zinc oxide DOX: Doxorubicin. Data are expressed as mean±S.D.

^a^ Significantly different between control and other groups at p<0.05 using scheffe test.

^b^ Significantly different between DOX and other groups at p<0.05 using scheffe test.

## Discussion

Results of both biochemical and histological investigations showed that DOX induced a marked reproductive toxicity through induction of oxidative stress that is protectable by nZnO administration. In this study, testicular and epididymal weight decreased by DOX administration in rats killed after 28 days. This weight reduction may be concerned on decrease of testosterone and LH levels (29). 

Furthermore marked decrease in reproductive organ weights by DOX can be result of reduced number of germ cells, atrophy of Leydig cells and lower rate of spermatogenesis, as confirmed by our histological findings of sperm density reduction in epididymis (26). In present experiment DOX caused a significant decrease in the testosterone and LH levels. Gamal Hozayen reported that the DOX-administered rats showed significant decrease of serum testosterone and LH concentrations (4). 

It has been reported that antineoplastic agents can disturb Leydig cells directly (31). Thus, the reduction in circulating testosterone is supposed to be resulting from a direct poisonous effect of DOX on the Leydig cells. Steroidogenesis in the male rats is stimulated by hypothalamic gonadotropin releasing hormone (GnRH) effecting to induce the production and release of LH, which binds to LH receptors on the membrane of Leydig cells to upregulate testosterone production (4). The reduction in LH level perhaps is result of damage in the negative feedback control of hypothalamic-pituitary axis (32). 

In addition, it is likely that the dysfunction of the pituitary in LH releasing was resulted from damage to the cell membrane-mediated signaling mechanisms involved in releasing LH. This conclusion is confirmed by the findings that DOX can interact with cell membranes and change biochemical functions of them, without entering the cells (33). So it seems that reduced LH level in our study is resulted from damaged pituitary cell membranes after DOX exposure. DOX-treated rats contained significantly higher levels of MDA in their plasma and lower levels of TAP, indicating the strong pro-oxidative actions of DOX. 

The adverse effects of DOX result mainly from its essential tendency to produce free radicals and block antioxidant enzymes in various tissues (34). The current study and other similar studies confirmed that lipid peroxidation, a downstream chain reaction started by free radicals, was triggered by DOX as revealed by the increased level of lipoperoxidation product, MDA (35). DOX voluntarily autooxidizes in the presence of oxygen, producing superoxide and consequently other ROS, which can stimulate lipid peroxidation (36). 

In the present study, epididymal sperm count decreased by DOX treatment while the number of dead and abnormal sperms increased and motility did not show significant difference. Howell and Shalet in 2001 showed that the incidence of male infertility following DOX chemotherapy resulted from changes in sperm parameters. Germ cells in testes are vulnerable to DOX-induced oxidative stress (37). Anthracyclines like DOX exert their antitumor properties as well as other organ toxicity by intracellular producing of free radicals and ROS accompanied by intercalation with DNA and consequent inhibition of topoisomerase (38). 

This increased oxidative stress effects on the sperm membranes, proteins and DNA (39-41). Therefore, DNA damage may be liable for the increased level of abnormal spermatozoa forms. As confirmed by acridine orange staining, treatment with DOX causes single/double strand breaks in sperm DNA. In the case of the adult rat testis, the gonadotoxic drug DOX induces programmed cell death in meiotic spermatocytes and type A and intermediate spermatogonia by intercalating into DNA to create strand breaks and by preventing topoisomerase II activity (7). 

These genotoxic alterations up-regulate expression of p53, an essential mediator of cell cycle stop considered to inhibit DNA replication in the presence of DNA damage, resulting apoptosis and finally drop in sperm counts (7). It is supposed that injured sperms are the source of free radicals. Free radical damage to developed sperms during their passage from the seminiferous tubules to the epididymes forms damaged or defected (amorphous, hook less, bicephalic, coiled, or abnormal tails) sperms as a major reason of male infertility (26). 

In response to some anticancer drugs like anthracyclines, the number of male germ cells undergoing apoptosis increases several folds. Because the most sensitive cells to DOX are the early spermatogenic cells and primary spermatocytes, treatment with DOX may lead to the loss of proliferating immature germ cells and finally of mature spermatozoa (1). 

Therefore, a possible reason for the disruption of spermiogenesis in the DOX-treated rats is failure of testosterone dependent attachment of spermatids to Sertoli cells (26). Zinc is a micronutrient existing in meat and seafood. Zinc finger proteins are involved in the genetic expression of steroid hormone receptors (42, 43). As found in the present study, Fuse *et al* in 1999 observed a relationship between zinc concentration and plasma testosterone level and indicated that sufficient seminal plasma content of zinc is necessary for normal sperm activity (44). 

Various studies have revealed the antioxidant and reproductive function of zinc (45, 46). Zinc salts have been shown to defend against oxidative harm and glutathione diminution in mice (47). This metal is vital in several features of male reproduction. Zinc levels are very high in the male reproductive system and seminal fluid (17). There is evidence that it is a scavenger of excessive O_2_- production by damaged sperm. Zinc plays an important role in the physiology of spermatozoa and spermatogenesis. 

It has also an essential role in spermatogenesis, sperm viability, prevention of sperm degradation and sperm membrane stabilization. It has been documented that zinc depletion causes atrophy of the seminiferous tubules and failure of spermatogenesis in rats. Zinc also possesses anti-apoptotic properties. Its deficiency can induce programmed cell death in various cell types whereas zinc supplementation can protect cells against different pro-apoptotic molecules, inhibiting apoptosis (17, 48, 49). Dawei *et al* in 2009 reported that nZnO is able to protect cell membrane integrity against oxidative stress damage, increase antioxidant enzyme levels and decrease MDA level (18). NZnO can improve antioxidant activity, enhance the activities of antioxidases and decrease the levels of free radicals (50). 

Recently, a number of studies have shown that nZnO is responsible for toxicity in some organs (21, 51, 52). Therefore, evaluation of the risk/benefits ratio for the use of nanoparticles in biology and medical developments seems reasonable (19). It is also noteworthy that nZnO shows cytotoxic properties in time- and concentration-dependent manners which are sometimes reversible (53-54). 

## Conclusion

In conclusion, our results suggest that reproductive toxicity induced by DOX is related to increased oxidative stress. Treatment with nZnO at the dose designed in this study improved reproductive dysfunction. It appears that nZnO is a potential antioxidant as an additive to chemotherapeutic drugs that are toxic to male organs. 

These results may provide further visions into proper treatment of male patients by improving spermatogenesis and sperm parameters*. *nZnO could be an effective adjuvant to enhance therapeutic efficiency and moderate gonadotoxic properties of DOX in clinical practice. However further studies are necessary to establish optimal doses of nZnO and receive the best safety profile.
